# Supplementary material for: Chromothripsis during telomere crisis is independent of NHEJ, and consistent with a replicative origin
Source: Genome Res. 2019 May;29(5):737–49. doi: 10.1101/gr.240705.118 (PMC6499312; doi:10.1101/gr.240705.118)
Supplement: Supplemental Material [file supp_gr.240705.118_Supplemental_file_1.zip › contigs/annotated_contigs/DB37/contig.2.DB37_length_673_mean_cov_7.07875185736.docx]

**DB37_length_673_mean_cov_7.07875185736**

```
TGTTTGTTTTTTTTTTTTTTCTTT|GCATGGTAGTGCATACCTGTTGTCCCAACTACTCAGATGGCTGAGGCAGGAGGATCAATTGAGC
                         >chr16:78765747-78765902 - E=2e-81
CCAGAGTTCAAGGCTGCAAAGAGCTGTGAAGGCACCACTGCACTCCAGCTTGGGCTATAAAGCAAGACCCTGTCTCTCAAAAAAAAAAA

AA|GAGTTCATCT|CTGTGTCAGAGATGAACAGAATACCCTGGTAATATCCCAATCTCACCAAGGCTGATCTGACAGAGCCCGATTACT
             >chr16:78742912-78743396 - E=1e-276
TGCAACAGGCTGGGCCCGCTGCCCAGGCCCTGTGAACCGGAATGGCCGAGAATCGCGGGCTTTCCTTCAGCCAGACAACTCCAACAAGC

TTCTCTCTGTGATCACTACGTAAGTTAAATGAGTTAATCTTGTTTTCCAGATGAACTGTTATTTTGTCTCCCGCAGGCAGAGATTACGG

GATAAAAGCATTGTGTTAACAGGAGGGCTGGGGGCTTTCCAGAGCCACATTCCACACACACAGAACCCCTCTCTCCAGTTCTTCCTCCC

ATTTCTATTTTTTCATGGATTTAGAGAACGAGGAGGAGAGTAGGGAACTTCAAAATGACACGCAGGGCGATAAACCTTTCAGATGAATG

ATGTCACTTCATAAGGGGTCAGGGGGCCAATTTATTTTCACGTTTCGAAACAT
```
